# Factors limiting youths’ practice of preventive measures toward the outbreak of COVID-19 in Oromia special zone surrounding Finfinnee, Ethiopia

**DOI:** 10.1371/journal.pone.0248495

**Published:** 2021-03-15

**Authors:** Zelalem Tadese Feyisa

**Affiliations:** Department of Sociology, Salale University, Fitche, Oromia, Ethiopia; University of Pennsylvania, UNITED STATES

## Abstract

**Background:**

Coronavirus disease 2019 (COVID-19) is a highly contagious viral infection, and it has negative effects on public health. The practice of preventive measures of the disease supports containment processes of the spread of coronavirus. However, the practice of preventive measures is affected by several associated risk factors.

**Objective:**

This study aimed to investigate the associated factors that limit the youths’ practice of preventive measures against COVID-19 in the study area.

**Methods:**

A community-based cross-sectional study was conducted. The study used a quantitative approach for collecting data from 384 youths using a survey method. Not practicing preventive measures was measured to determine whether or not youths applied hygiene practices, kept their distance, restricted their movements, and sought self-help or support in the past two months. Descriptive statistics were used to assess the distribution of study participants, and a binary regression model was executed to examine the association factors with inability to practice preventive measures with a p-value < 0.05 statistically significant.

**Results:**

Male youths (Adjusted Odds Ratio (AOR) = 0.06; 95% CI: 0.02, 0.16) were less likely to practice preventive measures. Older youth (AOR = 1.33; 95% CI: 1.13, 1.56), with higher education level (AOR = 1.03; 95% CI: 1.01, 1.06), and who had higher income (AOR = 1.34; 95% CI: 1.02, 1.78) were more likely to practice preventive measures. Further, the belief in the body’s immunity to resist the disease (AOR = 0.27; 95% CI: 0.11, 0.67), lack of paying attention to the disease (AOR = 0.07; 95% CI: 0.01, 0.73), ignorance of evidence to the disease (AOR = 0.31; 95% CI: 0.13, 0.74), ease of restriction of movements (AOR = 0.29; 95% CI: 0.12, 0.72), lack of sensitization to actions in the community (AOR = 0.39; 95% CI: 0.16, 0.96), and substance use (AOR = 0.11; 95% CI: 0.05, 0.21) were other factors that were inversely related to practicing preventive measures.

**Conclusions:**

The findings suggested that more intervention efforts, by either communicating to or reaching out all groups, should be employed. All segments of the population should be equipped with the facts that effectively support them practice preventive measures against the disease. Finally, the results suggested that youths should abstain from substance use, keep their distance in their pastime and avoid crowdings.

## Introduction

Coronavirus disease 2019 (COVID-19) is a highly transmittable and pathogenic viral infection [1–3]. The World Health Organization (WHO) declared that COVID-19 is a global pandemic caused by SARS-Cov-2 with a potential to spread frequently and rapidly from person to person through droplets on 11 March 2020 [2, 4, 5]. COVID-19 has multi-faceted negative effects on both public health and societal life, thereby attracting the attention of all nations [2, 6–8]. As a result, understanding the disease is an ongoing process and its clinical features are highly variable from mild to severe and fatal respiratory diseases [4, 9].

Globally, according to the Situational Report-114 of the WHO, 4.17 million COVID-19 confirmed cases and 3,000 deaths from the infection were reported on 13 May 2020 [10]. In Africa, the same report indicated there were 49,429 confirmed cases and 2,000 deaths from COVID-19. In Ethiopia, the Federal Ministry of Health in collaboration with the Ethiopian Public Health Institute reported the first COVID-19 case was confirmed in Addis Ababa, the capital of the country, on 13 March 2020 [11]. On 13 May 2020, the same report by the two agencies revealed that there were 261 confirmed COVID-19 cases and 5 deaths from it in Ethiopia [11].

Effective implementation of preventive measures are the primary tools in containing the spread of coronavirus at the community and individual levels [1, 7, 12, 13], since there are no proven vaccines and treatments against the disease at the time of writing this study. Preventive measures, such as hygiene practices, physical [social] distancing, movement restrictions, and seeking self-help or support, are effective tools in containing the spread of the virus [14].

A study conducted in Ethiopia by Kebede et al. [15] identified the young people were less likely to practice preventive measures, and in turn, such behaviors would be challenging for the containment processes of the virus in the future. Still another study conducted in Ethiopia [16] stated that the transmission rate of the disease is 4.08. Since the host of the virus is not yet known at the time of this writing, all individuals, including the young people, would be required to practice preventive measures. Owing to the transmissibility rate of the disease, the youths are at most risk if they do not practice preventive measures when they pass their time and their likelihood of getting infected with the virus and spreading it in the whole community is high.

Taking into account the likelihoods of youths’ adherence to the preventive measures is weaker, this study was carried out to inquire the associated risk factors affecting the youths’ adoption of preventive measures. The study was actually motivated by the following factors. Principally, this investigation was conducted based on the fact that the majority of Ethiopa’s population is young people and had the highest prevalence rate of COVID-19, and the infection was also first seen among young people. Secondly, since the environs of Addis Ababa (Finfinnee) constituted the study araes located within 30 km radius, there are frequent travels of the youth from the study sites to Addis Ababa (Finfinnee) and back to the sites for commercial activities [17]. Next, at the time of writing this work, all reported cases of the virus in Ethiopia were from Addis Ababa (Finfinnee) and the areas surrounding Addis Ababa (Finfinnee) are assumed to be highly exposed to the disease. Finally, the transmissibility rate of the disease, which is 4.08, was another motivation factor for conducting this inquiry [16]. As a result of such circumstances, this study had a keen interest in making an inquiry.

Recent existing literatures on COVID-19 precautions identify that adherence to the preventive measures of the disease is primarily affected by variables such as knowledge, attitudes, and perception [2, 12, 13, 16, 18, 19]. In fact, these studies took into account the limited facts about adherence to the practice of preventive measures. Other related studies [15, 20–24] indicated that useful knowledge, positive attitude, and thinking in appropriate ways toward the disease were not sufficient to contain its transmission. In addition, the aforementioned studies concluded that having a weaker adherence to preventive measures would be challenging to control the transmission of the virus.

An investigation of associated risk factors affecting individual’s inability to practice preventive measure is an essential tool for intervention purposes. In addition, it helps assess their association with adherence to the practices of preventive measures. Cognizant of such gaps in and the limited facts about the adherence to practice of preventive measures in the study area, this research aimed to assess the association of socio-demographic, sociocultural, environmental, and individual behavioral factors concerning youth’s inability to practice preventive measures for the disease.

## Methods and materials

### Study setting, design and period

The study was conducted in randomly selected six towns (Burayu, Sebeta, Sululta, Laga-Tafo, Gelan, and Dukem) from ten towns in Oromia Special Zone Surrounding Finfinnee (OSZSF). There were ten (10) towns that had similar attributes in this zone. Since all schools and youth recreational centers were in lockdown because of the COVID-19 outbreak, the researcher was forced to look for places where youths were actually available. Then, the researcher asked the heads of the offices of women, children, and youth of each selected town to find out where the youths in the towns actually spent their time. The study sites where youths actually spent their time in each selected town were the public places, such as small pieces of green belts, walking streets, public event squares, grocery stores, and patio door spaces. These sites were suggested to the researcher by all heads of the office. Public places are the focal points for sharing identities, concerns, and provide the only means of mutual access for individuals with diverse interests and backgrounds. A community-based cross-sectional study design was employed to assess risk factors associated with youths’ failures to practice preventive measures of the disease. The study was carried out from 15–25 May 2020 after two months of the first confirmed COVID-19 case was reported in Addis Ababa (Finfinnee), Ethiopia.

### Inclusion and exclusion criteria

The study participants were both young male and female residents of the study areas, who voluntarily took part in the study and who were able to read and write. Young people who were under the age of 16 and over 24 years of age and who lived in the study area for less than six months were not included in the study.

### Population and sample

The study was conducted among selected youths between the ages of 16 and 24. The study population were the youth who spent their time in public places. Thus, the samples were from different public places of each selected town.

### Sample size determination and sampling

A single population formula was used to determine the sample size. Accordingly, the formula for sample size determination used was: n = [p (1-p)] *[Z_α/2_)^2^/(e)^2^], where n denotes the sample size, Z_α/2_ is the reliability coefficient of standard error at 5% level of significance = 1.96, p represents the probability of youths who were unable to practice preventive measure of the disease (50%, no previous study found), and e refers to the level of standard error tolerated (5%) as stated by Hosmer and Lemeshow [25].

Based on this formula, it was determined that a sample size of 384 respondents would be sufficient to identify differences by respondent characteristics. The study also calculated the intracluster correlation coefficient (ICC) estimate (ρ) for the six towns based on the index of adherence to recommended preventive practice described below. The ICC was computed to be 0.018 (95% CI: -0.20–0.197), p-value = 0.429. A low ICC could reflect the lack of variability among the sampled clusters. In this study, the ICC value was small and also not significant, and thus the clusters were unlikely to account for the differences that were observed. Based on ICC (ρ = 0.018), the design effect (2.134), and power (80%), the minimum effective sample size (ESS) was computed to be 64 per cluster. Thus, for the 6 clusters, 384 total subjects were recruited with 64 per town.

A multistage sampling technique was used. Using the primary sampling units, random sampling was used to select six towns from the ten identified towns because all of the towns were also assumed to be having similar attributes in the practice of preventive measures. In the secondary sampling units, public places were selected randomly. The sample size was proportionally allocated to each selected town and public place. Finally, a simple random sampling was employed until the allocated sample size was reached.

### Reliability and pilot study

A preliminary phase was carried out to examine the reliability of a questionnaire for measuring preventive behavior before starting collecting primary data. Primarily, three experts from the field of infectious disease prevention and researches in Addis Ababa University, College of Medical Sciences and Health were invited as an evaluator to examine the degree to which the items in the questionnaire were relevant and could correctly measure the associated factors affecting youths incapability in practicing preventive measures. Next, questions inquiring individual behavior about the practices of preventive measures were modified to reflect both the outlook and actual practices. The next step was pretesting the reliability of the questionnaire using Cronbach alpha. Thirty (30) non-sampled respondents completed the 20-items questionnaire including individuals’ responses to questions that measured the practices of preventive measures using binary responses, 1 no response and 2 yes response. Based on these items and responses, alpha was computed to be 0.69 at Burayu, 0.73 at Sebeta, 0.70 at Sululta, 0.68 at Laga-Tafo, 0.72 at Gelan, and 0.74 at Dukem and these all values indicated the internal consistency of the questionnaire was high.

### Data collection and measures

The survey was conducted using tools that were adapted from WHO’s resources on COVID-19 [26]. The tools were developed in English and then translated to Afaan Oromo and Amharic, widely spoken languages in each selected district. The survey tools contained five components: 6 items of socio-demographic characteristics, 2 items of general information about the disease, 10 items of sociocultural measurements, 5 items of environmental measurements, and 5 items of individual behavioral measurements.

Trained BSc degree holders were recruited as data collectors and they were given intensive training on the objectives of the study and tools. During data collection, a minimum of one-meter distance was kept between data collectors and respondents and all of them wore an N95 surgical mask.

Practicing preventive measures was measured by variables such as hygiene practices, physical [social] distancing, movement restrictions, and seeking self-help or support [13, 15]. Hygiene practices include hand washing regularly with soap and water, avoiding touching the eyes, nose, and mouth with poor hands or fingers hygiene, covering the mouth and nose with a clean cloth while coughing and/or sneezing, and cleaning and disinfecting frequently touched objects and surfaces. Keeping physical [social] distance includes avoiding large gatherings, avoiding close contact with individuals who are sick, especially with flu or cold or fever or sneezing, and avoiding shaking hands with others; whereas, movement restrictions include restricting travel or movement and staying home if one is feeling sick or ill. Lastly, seeking self-help or support includes visiting the nearest hospital or health facility in case they get sick, purchasing medicines from the nearest drug store or pharmacy, and using a traditional treatment.

The study participants were asked about their experiences with all preventive measures over the past two months. Thus, the outcome variable, practicing preventive measures of COVID-19, was defined as:

$$Y=\left\{ \begin{array}{c} 0\;if\;youths\;not\;practice\;all\;preventive\;measures\;of\;COVID-19\;over\;the\;past\;two\;months\\ 1\;if\;youths\;practice\;all\;preventive\;measures\;of\;COVID-19\;over\;the\;past\;two\;months\; \end{array}\right.$$

Variables such as socio-demography, sociocultural, environmental, and individual behaviors were used as independent variables and all of them were answered with a yes/no response.

### Statistical analysis

The distributions of sampled youths were presented using descriptive statistics such as frequencies and percentages. The estimated effects of independent variables on adherence to the preventive measures were executed using a binary logistic regression analysis of odds ratio at 95% confidence intervals. All significant independent variables with p-value < 0.25 [27] in bivariate analysis were included in the multivariate logistic regression analysis. Furthermore, the Variance Inflation Factor (VIF) statistic was employed to test the existence of multicollinearity independent variables with a cut point value that was set to 10. The relative associations of independent variables with inability to practice preventive measures were interpreted using the adjusted odds ratio (AORs) at 95% confidence intervals. Only statistically significant independent variables were retained in the final model for the interpretation. The statistical significance level was set at a p-value < 0.05 (two-sided). Data analysis was managed using Statistical Package for Social Sciences (SPSS) version 20.0.

### Ethics approval and considerations

The study was conducted according to the principles of the European Commission for Social Sciences and Humanities Ethics and it fulfilled the requirements of Ethiopian National Health Research and Ethics Guideline. Although the Health Office of Oromia Special Zone Surrounding Finfinnee has no institutional ethics committee to review the survey, the study got an official permission letter numbered HOOSZSF/1248/12 from the Office. Consent responsibility for participation in the study by youths below the age of 18 years old was vested in either parents or legal guardians. Verbal informed consent was sought from every respondent. Moreover, data collectors were observed for 14 days after the completion of the survey. A potential risk was minimal at the time of the study.

## Results

### Socio-demographic characteristics of the study participants

As can be seen from Table 1, from a total of 384 youths who participated in the survey, more than two-thirds (69.5%) were males. More than three-fourths (77.1%) of the study participants were between the ages of 20 and 24. A quarter (25.5%) of the study participants were college and university graduates; 22.4% of them had a preparatory school educational level, and 17.8% of them had a high school educational level. Table 1 also shows the detail of other socio-demographic characteristics of the study participants.

**Table 1:** Socio-demographic characteristics of youths in OSZSF, 2020 (n = 384).

Variables	n	%
Sex		
Male	267	69.5
Female	117	30.5
Age (years)		
16-<20	88	22.9
20-<25	296	77.1
Mean(±St.D)	21.3(mean)	(±2.35)St.D
Educational level		
Be able to read and write	24	6.3
Junior Primary school (1–4)	45	11.7
Secondary primary school (5–8)	59	15.4
High school (9–10)	72	17.8
The preparatory school (11–12)	86	22.4
College and university	98	25.5
Median(mean) of educational level (in grades)	10(9.69)	
Religion		
Orthodox	125	32.6
Protestant	103	26.8
Islam	98	25.5
Waqefata	58	15.1
Occupational status		
Unemployed	113	29.4
Student	94	24.5
Employed	96	25
Self-employed	81	21.1
Monthly income (ETB^a^)		
≤500	88	22.9
501–1000	124	32.3
>1000	172	44.8
Median(mean)	550(599.3)	

a = Ethiopian Birr

### Sources of information on preventive measures against COVID-19

All study participants reported that they heard about preventive measures of the disease. The most common sources of information about the preventive measures that the study participants reported were the media such as television or radio (32%), family members either father, mother, brother or sister (22.8%), and social media like Facebook or Telegram (19.8%). Details of other sources of information are presented in Table 2.

**Table 2:** Source of information for preventive measures, OSZSF, 2020 (n = 384).

Variables	Categories	Source of information for preventive measures of COVID-19	
		n	%
Mass media	Television	71	18.5
	Radio	52	13.5
Social media	Facebook	58	15.1
	Telegram	18	4.7
Family members	Father	35	9.1
	Mother	30	7.8
	Brother or sister	23	5.9
Professionals	Health	21	5.5
	Musicians	15	3.9
	Artists	17	4.4
Stakeholders	Governmental	24	6.3
	**Non-governmental**	**20**	**5.2**

#### Indicators and responses to practices of preventive measures among youths

Two-thirds (66.7%) of the respondents were not wash their hands regularly with soap and water. In addition, 73.1% of the respondents were not use a face mask and were not cover the mouth or nose while sneezing or coughing. About 70.8% of the respondents were not avoided gatherings which were large. Detail of other practices of preventive measures were presented in Table 3.

**Table 3:** Indicators and responses to practices of preventive measures, OSZSF, 2020 (n = 384).

Variables	Responses of the study participants			
	No		Yes	
	n	%	n	%
**Practicing preventive measures of COVID-19**	**265**	**69**	**119**	**31**
** Practices 1: Hygiene practices**	**72**	**70.6**	**30**	**29.4**
Hand-wash regularly with soap or water	16	66.7	8	33.3
Avoid touching (eyes, nose, mouth with hands not washed)	20	69	9	31
Use a facemask and cover the mouth or nose while sneezing/coughing	19	73.1	7	26.9
Disinfect the frequently touched objects	17	73.9	6	26.1
** Practices 2: Keeping physical [social] distance**	**67**	**70.5**	**28**	**29.5**
Avoid large gatherings	17	70.8	7	29.2
Avoid close contact with individuals who is sick with flu cold	23	71.9	9	28.1
Avoid shaking hands with others	27	69.2	12	30.8
** Practices 3: Movement restrictions**	**55**	**67.9**	**26**	**32.1**
Restrict travels or movements	34	70.8	14	29.2
Stay at home if felt sick or illness	21	63.6	12	36.4
** Practices 4: Seeking self-help/support**	**71**	**67**	**35**	**33**
Visit the nearest hospital or health facility when getting sick	21	61.8	13	38.2
Purchase medicines from a drug store or pharmacy	24	75	8	25
**Use traditional treatment**	**26**	**65**	**14**	**35**

### Bivariate analysis of factors and practices of preventive measures

The bivariate analysis of each independent variable with the outcome variable was employed to identify the association between them. Accordingly, sex, age, educational level, income level, confusion about the disease, perception the body’s immunity to resist the disease, belief in the disease being found only in the capital city, lack of consensus toward the disease at home, lack of attention to the disease by community, ignorance of facts toward the disease, lack of trust on early evidence toward the disease, lifestyles with depression, ease restriction of movements, absence of mass screening for the disease, lack of sensitization to action in youths’ settings, substance use (alcohol, khat, smoking), and negligence toward practicing preventive measures were significantly associated with the outcome variable at p-value < 0.25. All significant independent variables in the bivariate analysis were included in the multivariate logistic regression analysis. Details of the analysis are presented in Table 4.

**Table 4:** Logistic regression predicting adherence to preventive measures by youths, OSZSF, 2020 (n = 384).

Limiting Factors	Be able to practice preventive measures		COR (95% CI)	AOR (95% CI)
	No	Yes		
**Socio-demographic**	**n (%)**	**n (%)**		
**Sex**				
Male	226(84.6)	41(15.4)	0.12(0.06,0.24) *	0.06(0.02,0.16) **
Female	39(33.3)	78(66.7)	1	
**Age (year)**	-	-	1.16(1.05,1.28)*	1.33(1.13,1.57)**
**Educational level**	-	-	1.05 (1.03,1.07)*	1.03(1.01,1.06)**
**Income level (Ethiopian birr)**	-	-	1.27(0.99,1.67)*	1.34(1.02,1.78)**
**Socio-Cultural Factors (yes)**				
Pastime activity outside home	99(79.8)	25(20.2)	0.84(0.46,1.54)	0.79(0.32,1.99)
Being a daily laborer	63(70.8)	26(29.2)	0.82(0.44,1.51)	1.05(0.41, 2.72)
Confusion about COVID-19	139(65)	75(35)	1.84(1.17,2.88)*	0.84(0.34, 2.06)
Perception the body’s immunity to resist the disease	178(62)	109(38)	0.99(0.62,1.56)*	0.27(0.11,0.67)**
Belief in the disease being found only in the capital city	109(67.3)	53(32.7)	1.25(0.78,2.03)*	0.47(0.14,1.56)
Lack of consensus toward the disease at home	117(69.2)	52(30.8)	1.11(0.60,2.04)*	0.47(0.18,1.26)
Lack of attention to the disease by community	198(90.8)	20(9.2)	0.41(0.18,0.96)*	0.07(0.01,0.73)**
Perception of COVID-19 is a government issue	56(70)	24(30)	0.90(0.49,1.68)	1.10(0.46,2.67)
Ignorance of facts toward the disease	187(65.6)	98(34.4)	0.11(0.02, 0.86)*	0.31(0.13, 0.74)**
Use of home remedies for prevention purposes	116(51.6)	109(48.4)	0.87(0.56,1.36)	1.14(0.59, 2.20)
Perception as it is prevented by eating foods frequently	82(62.1)	50(37.9)	0.80(0.49,1.30)	1.17(0.57, 2.37)
Lack of trust on early evidence toward COVID-19	112(86.2)	18(13.8)	0.86(0.51,1.47)*	0.89(0.41, 1.94)
Lifestyles with depression	122(75.3)	40(24.7)	1.15(0.70,1.91)*	0.85(0.40,1.81)
**Environmental Factors (yes)**				
Ease of movement restriction	211(79.6)	54(20.4)	0.55(0.34,0.89)*	0.29(0.12, 0.72)**
Absence of mass screening for COVID-19	71(83.5)	14(16.5)	0.41(0.18,096)*	0.78(0.36, 1.73)
Lack of centers for information toward COVID-19	29(72.5)	11(27.5)	0.40(0.10,1.59)	0.83(0.11, 6.30)
Lack of sensitization to actions in youths’ settings	182(75.2)	60(24.8)	1.03(0.59,1.83)*	0.39(0.16, 0.96)**
Lack of a confirmed case at nearby or within a locale	103(59.2)	71(40.8)	2.41(1.04,5.60)	1.38(0.42, 4.52)
**Individual behavior (yes)**				
Substance use (alcohol, khat, smoking)	186(70.2)	79(29.8)	0.09(0.05,0.16)*	0.11(0.05,0.21)**
Negligence toward practicing preventive measures	127(64.5)	70(35.5)	0.83(0.51,1.33)*	1.08(0.51, 2.30)
Responsible as a messenger for family members	20(28.2)	51(71.8)	0.45(0.17,1.23)	0.47(0.11, 2.03)

*significant at p<0.25

** significant at p<0.05

COR: crude odds ratio, AOR: adjusted odds ratio

### Goodness fit of the model

Multicollinearity test indicated VIF value of each independent variable was less than 10, showing that the collinearity among the independent variables was weak. The Hosmer and Lemeshow (χ^2^ = 14.907, p-value = 0.061) and the likelihood ratio test (χ^2^ = 206.272, p-value<0.001) indicated that the logistic regression model fits the data well. Thus, the study rejected the null hypothesis, which states that there was no difference between the model without explanatory variables and the model with explanatory variables. Furthermore, when the ratio of Pearson chi-square statistics to degrees of freedom was nearer to 1, the logit model adequately fits the data, and vice versa. In the current study, the result of Pearson chi-square showed that the ratio was equal to 1.040, indicating that data fitted the model, and a logistic regression model can be established. The larger the value of Cox and Snell R^2^ (0.416) and Nagelkerke R^2^ (0.585) indicates higher accuracy and the model was acceptable.

#### Factors limiting youths’ practice of preventive measures

Multivariable binary logistic regression analysis was executed to assess the associated risk factors that limited the youth to practice preventive measures, and the results were presented in Table 4. Male youths were 94% times less likely to adhere to preventive measures compared to female youths (AOR = 0.06; 95% CI: 0.02, 0.16). As the age of youths increase by a unit, the youths’ adherence to preventive measures increased 1.33 times (AOR = 1.33; 95% CI: 1.13, 1.56); whereas an increase in the youths’ educational level was associated with a 1.03 times more likely adherence to preventive measures (AOR = 1.03; 95% CI: 1.01, 1.06). Youths with a higher income per month were 1.34 times more likely to adhere to preventive measures (AOR = 1.34; 95% CI: 1.00, 1.78).

Youths who believed that the disease was resisted by the body’s immunity were 73% times less likely to adhere to preventive measures compared to youths who did not believe that the disease was resisted by the body’s immunity (AOR = 0.27; 95% CI: 0.11, 0.67). On the other hand, youths who lived in the community and did not pay attention to the disease were 93% times less likely to adhere to preventive measures compared to youths who lived in the community and paid attention to the disease (AOR = 0.07; 95%CI:0.01, 0.73). Youths who ignored evidences of the disease were 69% less likely to adhere to preventive measures compared to youths who were aware of the disease (AOR = 0.31; 95% CI: 0.13, 0.74). Moreover, youths who lived in settings where there was an ease of movement restrictions were 71% less likely to adhere to preventive measures compared to those who lived in lockdown settings (AOR = 0.29; 95% CI: 0.12, 0.72).

Youths who resided in the community where there was a lack of sensitization to actions were 61% less likely to adhere to preventive measures compared to youths who resided in the community where there were sensitization to actions (AOR = 0.39;95% CI: 0.16, 0.96). Additionally, those youths who involved in substance use were 89% less likely to adhere to preventive measures compared to youths who were not involved in substance use (AOR = 0.11; 95% CI: 0.05, 0.21).

## Discussion

The current study found that the prevalence of not practicing the preventive measures of the disease was high (69%) among the study participants in the study area. However, all the study participants claimed that they heard preventive measures of the disease from various sources, such as media platforms, health professionals, and government officials.

The results showed there were gender differences in practicing preventive measures where male youths were less likely to practice preventive measures compared to female youths. The result was consistent with the findings of previous studies conducted in Bangladesh [2], Iran [18], and China [19]. These studies identified male youths had less knowledge than female youths about the disease. However, the reasons for the males’ lower level of knowledge and lack of awareness of the disease were not illustrated very well. Following the onset of the outbreak, facts or information concerning the disease was accessed by using media platforms. In a male-dominated society like Ethiopia, males are involved more in activities taking place outside of the home. Moreover, their activities are characterized by risky behaviors. Nevertheless, females are more involved in domestic activities, including serving and caring for the whole family. Owing to discrepancies between females and males in their social roles, access to information from media platforms between them are different. Along this line, the action of equipping both male and female youths with facts concerning the disease may have been quite different. Because of such differences, male youths may have had less information about how to practice preventive measures than female youths, and this should be studied further. Since there is no proven vaccine against COVID-19 or drug to cure it is developed yet, the best way to minimize the spread of coronavirus is by maximizing how all individuals get insights that would help them take precautions.

The study also revealed that younger youths did not practice preventive measures compared to older youths. The result was consistent with previous studies carried out in Egypt [1], Jimma [16], and China [19] following the outbreak. The aforementioned studies revealed that young people who did not practice preventive measures had a lower level of knowledge. The current study argued not only a lower level of knowledge, but also other variables played pivotal roles in limiting the youths’ practice of preventive measures. It is quite common in the study area that young people depend on their families; hence, the youth may need numerous instructions from parents and other relevant authorities so that they can deal with sudden events or outbreaks like COVID-19. Dependence on and seeking a lot of support from family on how to practice preventive measures may be considered as another limiting variable for the practices of preventive measures among young people. Moreover, the lack of experience in how to practice preventive measures and an absence of reports of confirmed COVID-19 cases seem to limit the youths’ taking of preventive measures at a very young age. Factors that have a potential to limit youths in practicing preventive measures might be a misconception of the disease. Thus, for such limiting factors, educating whole family members on how to practice preventive measures is highly needed.

It was found that youths with lower educational levels did not practice preventive measures. The result was consistent with studies conducted in India [13], Uganda [20], and Asia [21] for assessing the knowledge level of individuals with the disease. It is evident that individuals with higher educational levels are more likely to understand sudden public problems like the outbreak. Moreover, these individuals have better efforts in managing complex issues related to the disease compared to individuals with lower educational level. These factors may be responsible for the youths’ involvement in risky behaviors and the factors should be studied further. In addition, the youth were unable to search for additional information that could help them practice preventive measures. Hence, to accomplish the effective interventions and containments, health education campaigns by health workers and other concerned bodies are significant steps.

The result indicated that youths who had lower income per month did not practice preventive measures than youths who had higher income per month. The result was consistent with studies conducted in Egypt [1], and Peru [29] to assess the practice of preventive measures. Individuals with lower income per month are individuals who are also with lower level of education [32]. Having or not having resources, including access to information, may have been determined by income level. Since the disease is a novel disease and broke out suddenly, a lot of people are rushing to get a detailed insight from the Internet [29]. Nevertheless, such information was not accessible to individuals with lower income and education level. It is evident that individuals with low income are mostly involved in low-paid jobs. Moreover, for survival reasons, these individuals can not be staying home, i.e., they can not saty home without earning their daily bread. Because of the scarcity of resources, they might be involved in risk behaviors and be unable to buy personal protective equipment. The containment goals of the disease are met if all individuals are fighting together against the spreading of the disease. Hence, supporting individuals with lower income by providing them with necessary preventive measures is essential for the containment of coronavirus.

Because youths perceived that the disease was resisted by body’s immunity, they not practicing preventive measures. The present result was consistent with studies conducted in Italy [5], Jordan [12], and Myanmar [15] to assess the risk perception of the disease. It is evident that young people who live with their families are dependent. For this reason, they may be less stressed about problems affecting individuals’ well-being and a health problem like COVID-19. Consequently, young people are in a good state of health both physically and mentally. In addition, they are less involved in risky behavior compared to those youths with stressful events. Following the outbreak, rumors concerning the disease revealed that young people were at less risk of death, asymptomatic, and less infected with coronavirus than adults. Thus, such observations or beliefs may have influenced the youth to perceive that their body immunity helped them in resisting the infection. Currently, WHO and recent investigations confirmed the coronavirus has a potential to infect all human beings [28]. Regarding such issues, it was crucial to equip youths with facts on coronavirus potential of infecting all individuals.

Paying less attention to the disease in the community was also another variable that limited youths’ practice of preventive measures. The result was consistent with previous studies conducted in Asia [21], Peru [29], and China [30] which underlined individuals’ behaviors with facts about the practice of preventive measures. The results of the current study argued that not only individuals’ actions, but also where they live, including the community and environment, may determine either their patterns or natures of practicing preventive measures. Variables such as lack of psychosocial changes among populations, lack of preparedness to practice preventive measures, and being passive to the disease by community may have influenced the youth to pay less attention to the preventive measures. The absence of reactions between social and psychological factors, such as anxiety, stress, and emotional changes due to the fatal nature of the disease, may have made the community pay less attention to the practice of preventive measures. Thus, greater empowerment of communities with insights that can change their views on how to practice preventive measures for the containment processes may be needed.

The results indicated that ignorance related to facts on the disease was another variable that was associated with youths’ practice of preventive measures. The result was consistent with studies conducted in India [6], Pakistan [24], and Florida [31] to assess the practice of preventive measures. These studies revealed that lack of awareness and less knowledge in the preventive measures resulted in individuals not practicing preventive measures. However, these studies did not explain why individuals had a lower knowledge and were not aware of the preventive measures. This study tried to identify why youths were unaware of preventive measures. An individuals’ inability to gain knowledge of the preventive measures may be due to lack of personal experience and absence of continued education. In addition, other reasons might include lack of consciousness raising events like presenting patients who recovered to the media, thereby clearing confusion on how to apply preventive measures. Since the disease is a novel disease, confusion about the disease may result in the youths’ behaving without sensing the preventive measures. Thus, a lack of gaining knowledge and insight through a direct observation or participation might limit youths’ practice of preventive measures. In this regard, equipping the community with facts through continued public education is an essential tool in the containment process.

A lack of sensitization to actions on how to practice preventive measures in the youths’ settings was another variable that limit youths’ practice of preventive measures. This result was consistent with studies conducted in Bangladesh [2], and Ethiopia [11] to assess the practices of preventive measures. In Ethiopia, various media platforms were used as sensitization tools, although the young males and females responded ineffectively to the preventive measures as there was a lack of sensitization to action in their settings. The act of sensitization with the practice of preventive measures seemed to be done superficially. In other words, the actions may be done in line with collective norms, values, and traditions and the actions did not address a specific segment of the population like youths. Thus, a sensitization to action may not be carried out in a cultural context of every segment of the population for a contagious infection like COVID-19, and this would make the containment procedures and rules challenging.

Following the COVID-19 outbreak, an easy of restriction of movement that was imposed in Ethiopia made the youths not practicing preventive measures. The result was consistent with the findings of studies conducted in Bangladesh [2], Ethiopia [11], and the Provinces of Kabul, Kunduz and Khost [22] to assess the practices of preventive measures. The aforementioned studies indicated that the type of movement determined the effectiveness of public health interventions and containment procedures. An easier movement of people with a little worry about the preventive measures may result in a weaker adherence to the preventive measures. Following the outbreak, Ethiopia has declared a state of emergency and introduced ease restrictions on services, such as public transportation, marketplaces, banking, and kindly requested residents to stay home and not to leave home without an emergent need. Since the disease is a newly emerging case, it is advisable if all people take the necessary and effective preventive measures against the disease as much as possible.

Involvement in substance use was associated with less practice of preventive measures. Substance use, such as alcohol use, khat use, and cigarette or hashish use in groups, are commonly practiced in Ethiopia. Use of substances in a group was found to be a risky behavior and a high-risk practice for the well-being of individuals [32, 33]. Drawing upon ideas stemming from such assumptions, individuals who involved in any risky behaviors and high-risk conditions are less likely to be able to practice preventive measures against contagious infections like COVID-19. The result was consistent with previous studies conducted in India [6], Italy [8], and Ethiopia [16] to assess the attitudes and perceptions individuals had about the disease. The main reason that makes youths’ substance use a risk potential is their inability to practice preventive measures in the places where they actually used substances. Young people normally involve in substance use outside their home with others in groups. In other words, the places where substances are used are croweded, and young people are with less attention to physical distance.

### Study strengths and limitations

The study aimed to assess the associated risk factors that were associated with youths’ failures to practice preventive measures against COVID-19. One strength of this study was the questionnaires in the survey were developed by experts and they had high reliability. In addition, the selection of various potential factors that might be used to assess preventive measures was sensitive to identify factors that were related to lack of preventive practice among youth.

The major limitation of the study were the study participants might be afraid of reporting the right information for the reason that they would be punished because they failed to follow rules and protocols of COVID-19 preventions. Another limitation was that the accuracy of the data was limited due to the use of a self-report survey, although it was considered to be a valid method of evaluating the practices of preventive measures. The final limitation was related to the statistical analysis where the large number of comparisons used in the current study may lead to some false-positive findings. Therefore, the current results should be interpreted with caution.

## Conclusion

The current study contributed to research into the risk factors associated with youths’ failures to practice preventive measures for COVID-19. Lower practice to preventive measures was observed among younger, male, less educated youths, and lower-income level youths. To be able to practice the preventive measures of the disease, more intervention efforts using different tools should be used by either communicating to or reaching out to these groups. Although the government and stakeholders have taken significant steps to limit the spread of the disease, more efforts should be needed for equipping all segments of the population with the facts about the disease. There was no proven vaccine or treatment for the disease; hence, a high level of understanding on how to practice preventive measures must be achieved in the community to stop the spread of the virus. Lastly, youths should refrain from risky behaviors, such as involvement in substance use, social gathering for passing time, and join the crowded places. The author recommended for future studies to incorporate more variables to identify factors that can result in more adherence to the preventive measures of the disease.

## Supporting information

S1 Data (SAV)

## Abbreviations

COVID-19: Coronavirus disease 2019
OSZSF: Oromia Special Zone Surrounding Finfinnee
WHO: World Health Organization
